# A space-gamified approach to examine muscle contraction behaviour in children and adolescents with spastic cerebral palsy: feasibility, acceptability and repeatbility

**DOI:** 10.3389/fped.2025.1520162

**Published:** 2025-06-05

**Authors:** Jule Heieis, Ibrahim Duran, Eckhard Schönau, Christoph Fritzsche, Bettina Götz, Laura Kehe, Moritz Meier, Karoline Spiess, Wilhelm Bloch, Jörn Rittweger

**Affiliations:** ^1^ Department of Muscle and Bone Metabolism, Institute of Aerospace Medicine, German Aerospace Center (DLR), Cologne, Germany; ^2^ Section Molecular and Cellular Sports Medicine, Institute of Cardiology and Sports Medicine, German Sports University, Cologne, Germany; ^3^ Department of Pediatrics and Juvenile Medicine, University of Cologne, University Hospital Cologne, Cologne, Germany; ^4^ Department of Pediatrics and Juvenile Medicine, UniReha Center for Prevention and Rehabilitation, Cologne, Germany

**Keywords:** cerebral palsy, muscle, spasticity, contraction, muscle control, gamification, biofeedback, neurorehabilitation

## Abstract

**Introduction:**

Cerebral palsy (CP) is the most common disease affecting mobility among children. However, relatively little is known about the muscle phenotype and the resulting impairments in muscle function of this population. We therefore examined feasibility and acceptability of a muscle testing protocol that is based on the muscle examinations of astronauts and in bed-rest studies in children and adolescents with CP (clinical trial registry number DRKS00031107).

**Methods:**

Twelve participants, aged between 8 and 18 years, with CP and age-matched able-bodied counterparts (Ctrl) have been included to the study. They completed testing procedures on two visits. Participants performed isometric maximum voluntary contractions, step and ramp contractions in plantarflexion on a custom build dynamometer. The tasks were visualized using a torque-controlled video game. We computed steadiness, defined as standard deviation of the fluctuations, and slope, as well as the achieved MVC. Data were statistically analyzed via Intraclass correlation coefficient (ICC) for between-visit analysis and Mann-Whitney U test for between-group analysis.

**Results:**

One participant of the CP group was not able to perform the tasks and dropped out for the second visit. Especially younger children and children with cognitive impairments were not able to adequately answer the acceptance questionnaire. The MVC of Ctrl was higher in both visits and was excellently repeatable. During step contractions Ctrl showed lower fluctuations in both visits. Also, during ascending ramp contractions Ctrl showed less fluctuations but only at visit 1. During descending ramp contractions steadiness was better in Ctrl at both visits. Performance parameters were all poorly repeatable, because the CP group improved their performance in all tasks at visit 2.

**Discussion:**

Application of our gamified muscle testing protocol was well acceptable and mostly feasible. Contrasting with constant isometric contractions and decreasing ramp contractions, the performance of children with CP during ascending ramp contractions improved to the level of control subjects within 2 visits. A crucial prerequisite to perform successful measurements are good cognitive skills and at least one familiarization visit.

**Clinical Trial Registration:**

https://www.drks.de/DRKS00031107, identifier (DRKS00031107).

## 1 Introduction

Spasticity occurs secondary to lesions that affect the information stream from the upper to the lower motor neurons, as for example in persons affected by cerebral palsy (CP). Accordingly, these patients suffer from functional impairments in activities of daily living. Not all impairments can solely be explained by neural deficits. Secondary to the neuronal impairment, changes in the spastic muscle have been found. Cerebral palsy leads to decreased muscle belly length (1, 2), volume (1–4), cross-sectional area and thickness (3, 5–7), which correlate with decreases in muscle strength. The published literature is highly inconsistent with regards to effects on fiber pennation angle and length. At macroscopic level, the spastic muscle fiber is passively stiffer than the non-spastic muscle fiber. At single-fiber level, this is explained by alterations of the titin protein (8). Interestingly the exaggeration in passive stiffness becomes blunted at the level of fiber bundles, most likely due to the unorganized structure of the extracellular matrix (9). It has been found that changes in muscle architecture due to spasticity correlate with performance of different functional tasks (10). However, little is known about the direct impact of all these changes in muscle morphology on intramuscular strain-stress behaviour and function. Investigation on muscle function typically uses a testing protocol involving voluntary and controlled contractions. Based on the muscle examinations of astronauts and in bed-rest studies we created a measurement setup with a testing protocol including the essence of different examination procedures. This protocol includes the testing of three different isometric plantarflexion contractions—maximum voluntary contractions (MVC), ramp contractions and step contractions. Investigating plantar-flexors is of great interest because of the high prevalence of equinus (11) and the early manifestation of increased muscle stiffness within these muscles (12) in children suffering CP. Additionally, the triceps surae muscles play a key role during gait by controlling balance and velocity (13). Maximum voluntary contractions can be used to investigate muscle strength and are the basis for step and ramp contractions. Performing step contractions makes time-consuming measurements like muscle oxygenation or sonographic elastography possible. Ramp contractions on the other hand allow high resolution ultrasound imaging of muscle contraction at the muscle’s mid-belly to investigate fascicle kinematic or at the muscle-tendon-junction to measure tendon elongation and stiffness. Both contraction types can be used to analyse electromyographic signals of agonists and antagonists to investigate muscle activation patterns. Clearly, voluntary contraction assessments have potential not only for science but also for clinical application in cerebral palsy. However, it is not clear how well these patients can really perform the aforementioned types of voluntary contractions, given their specific disability. Therefore, we examined feasibility, repeatability and acceptability of our measurement setup and protocol in children and adolescents with CP and able-bodied controls.

## 2 Materials and methods

### 2.1 Study design

To judge feasibility and acceptability, the study was designed as parallel-group design with a group of CP patients and an age-matched group of able-bodied counterparts. To asses reproducibility, two visits were planned on separate days for each participant. The study setting was the UniReha’s Queen Rania Rehabilitation Center in Cologne. Prior to study commencement, it had been approved by the ethics committee of the university hospital cologne and registered with the German Register for Clinical Studies (DRKS) with the index DRKS00031107 (www.drks.de/DRKS00031107).

### 2.2 Participants

All participants gave informed, written consent before participation. In case of underaged participants, informed and written consent was obtained from their parents. The patient group consisted of twelve adolescents between 8 and 18 years with diagnosed unilateral or bilateral spastic cerebral palsy (CP). Participation was offered to the adolescents with CP upon recommendation of the treating physical therapists based on the patient’s cognitive and motor skills. Exclusion criterion was reduced knee and ankle joint mobility inhibiting the measurement position of $90^{\circ }$ knee flexion and neutral ankle position. For each patient healthy participants with matching sex and age (±1 year) were recruited for the control group.

### 2.3 Measurement setup and protocol

**Figure 1:**
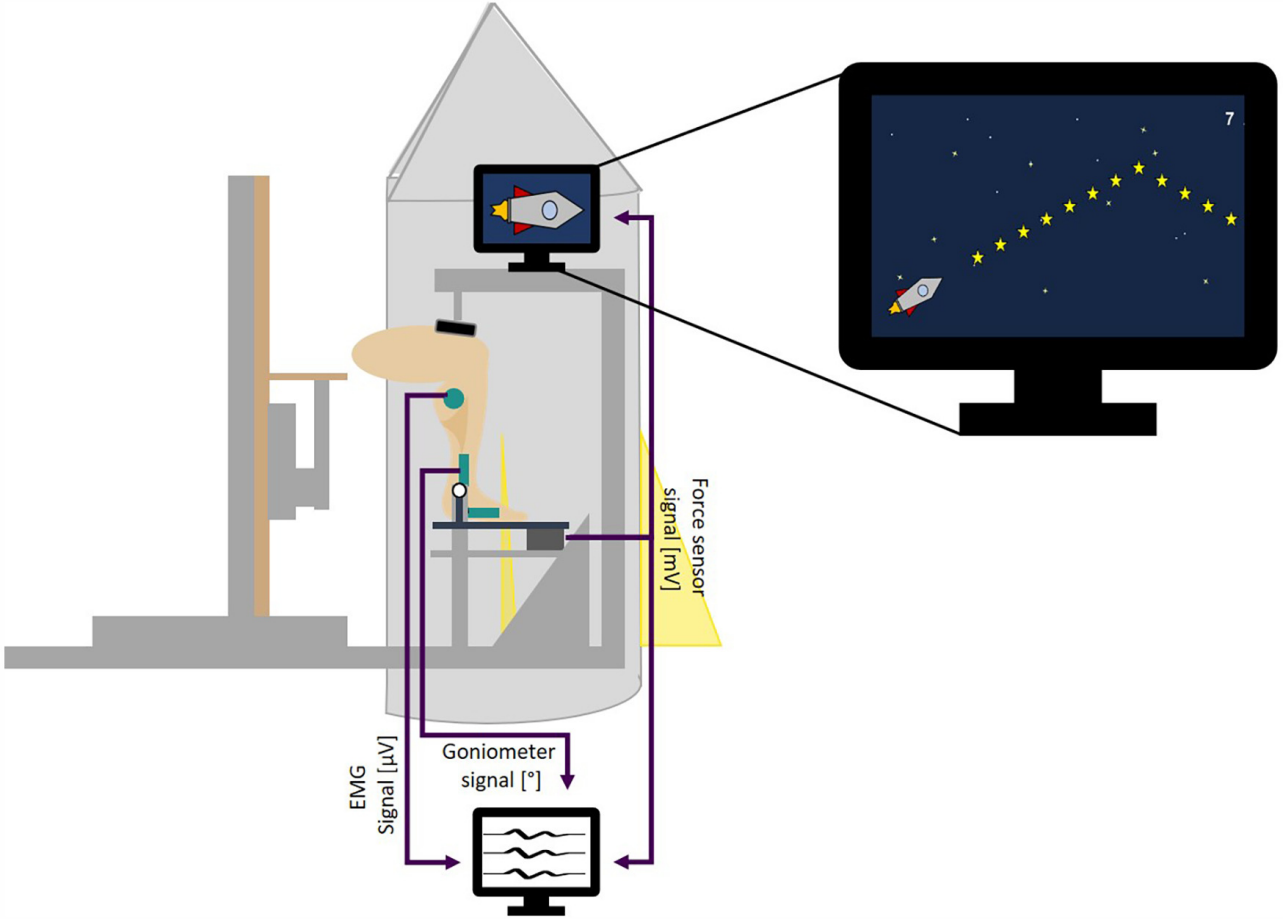
Measurement setup. Force, EMG and goniometer signals are collected simultaneously. The force signal is additionally transmitted to control the video game.

The custom-built measurement device consisted of a adjustable seat and a fixed dynamometer (Figure 1). The dynamometer’s working principle was based on a foot plate that was rotatable around a fixed axis of rotation (AOR) and rested on a force sensor under the front foot. The participants’ medial malleolus was aligned with the dynamometer’s AOR. To achieve the anticipated measurement position, the seat platform’s height above the AOR was adjusted to the length of the shank (distance from medial malleolus to medial knee joint cleft). The distance from the seat to the dynamometer was adjusted until the shank was oriented vertically (neutral ankle joint angle). The heel was prevented from lifting during force generation by stabilizing the distal thigh from the ventral side by a padded metal bar, serving as resistance (Figure 1). A soft foam pad in front of the foot served as landmark for the foot position, in case the foot has been moved between trials. The foot has not been fixated further to allow motion in case of arising spasms. To increase acceptability and compliance of the participants the whole measurement was gamified. The dynamometer part of the measurment device was covered in a rocket-style aluminium shell and all tasks were visualized in a torque controlled video game in which a digital rocket rose up with increasing plantarflexion torque.

There were three tasks given with three trials per task, each of them demanding different types of isometric plantarflexion contractions. In participants with unilateral CP, the affected leg was chosen, while the leg with better movement was chosen in patients with bilateral CP. The control group participants’ right leg was examined. The first task was the maximum voluntary isometric contraction (MVC). Participants were asked to drive the game rocket as high as possible, meaning to physically exert their largest possible torque. During trial 2 and 3, the maximum previously achieved torque was always displayed. From the overall maximum achieved, submaximal levels were defined at 20%, 40%, and 60% MVC. In three different trials these levels were presented for 16 s as horizontal stars in the second task “step contraction.” To accomplish this task, participants were asked to collect 30 stars, that were arranged in a step-wise fashion, with five stars at rest, followed by 20 stars at the desired contraction level, back to five stars at rest. During the third and last task “ramp contractions” participants performed continuously increasing and decreasing isometric contractions for eight seconds each from rest to 100% MVC and back. This task was visualized by stars arranged in a triangular shape (Figure 1).

The repeatability measurements were taken after an interval of at least 24 h.

### 2.4 Data acquisition

At the beginning of the first visit height and weight data, as well as the Achilles tendon lever arm (ATLA) length were collected. Afterwards the participants have been equipped with electromyography (EMG) electrodes at the soleus, lateral gastrocnemius and tibialis anterior muscle according to SENIAM standards (www.seniam.org) and with a goniometer (Noraxon U.S.A. Inc., Scottdale, United states) at the ankle joint. Force, EMG, and goniometer signals as well as a trigger signal of a custom-built hand trigger have been transmitted to a Noraxon system (Noraxon U.S.A. Inc., Scottdale, United states) and recorded during the whole measurement using the software myoResearch®version 1.08 Master Edition and a sampling rate of 1,000 Hz. The hand trigger was pressed at the beginning and end of each trial.

### 2.5 Data processing

All data processing has been performed using the programming language R version 4.3.2 (www.r-project.org) with the RStudio development environment (14). Force and joint angle signals were filtered using a fourth order Butterworth low-pass filter with a cutoff frequency of 5 Hz. The force signal was converted to Nm via calibration with a 1 m lever and a 10 kg mass. Afterwards the data files of each subject cut into individual trial recordings with the hand trigger signals. Resting torque was defined in each of the three MVC trials as mean torque of a manually defining 200 ms period before contraction with no visible contraction in the EMG signals. The MVC torque of each trial was calculated as average torque of the torque plateau. The plateau was defined as the area where the torque reached over 90% of the maximal torque. The maximal MVC value out of the three trials was then set as total MVC to calculate the contraction levels for further trials of tasks 2 and 3. For all tasks performance parameters have been calculated and statistically analyzed. In case of comparable performances between groups, additional muscle parameters about fascicle mechanics and muscle activation have been analyzed.

#### 2.5.1 Performance assessment

**Figure 2:**
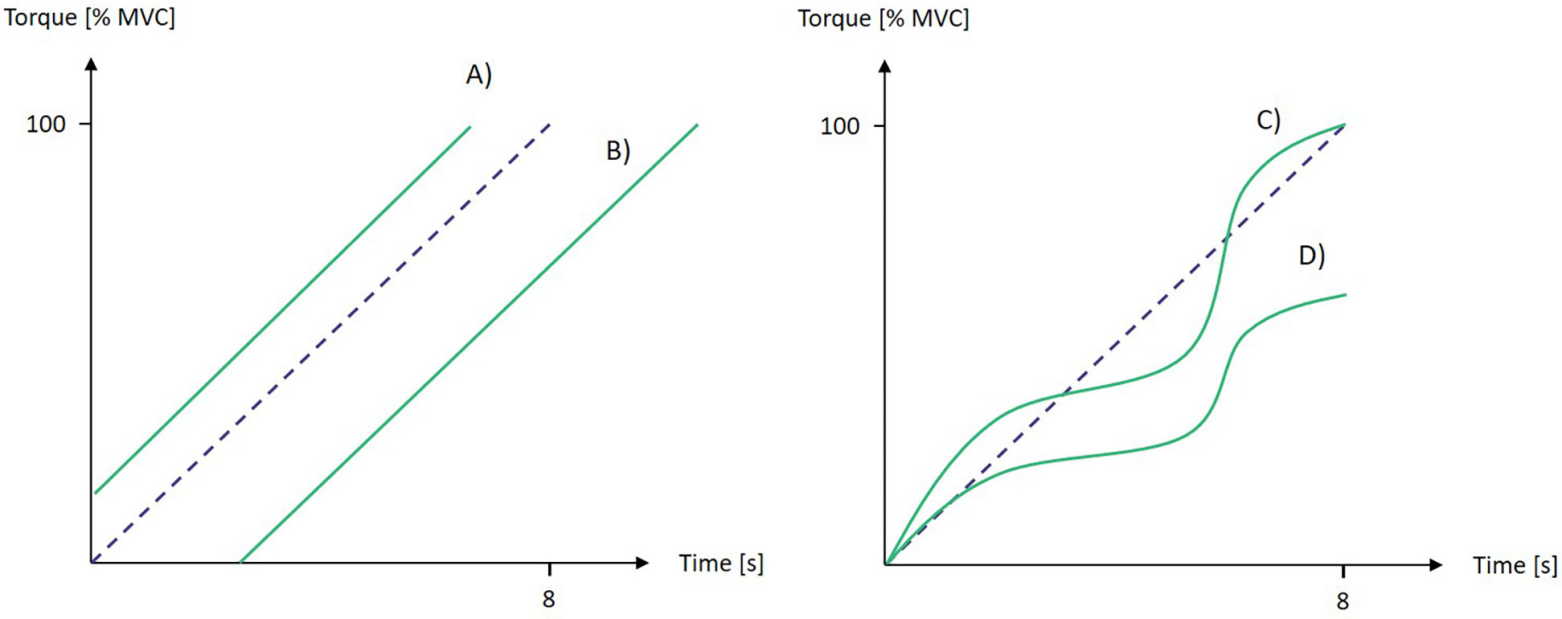
Influence of exemplary torque graphs (red lines) during ascending ramp contractions on performance parameters slope and steadiness. The blue dashed line indicates the prescribed path. **(A)** perfect steadiness and slope. **(B)** perfect steadiness and slope. **(C)** poor steadiness but perfect slope. **(D)** poor steadiness and slope.

To quantify the performance in tasks 2 and 3, the torque parameters steadiness and slope have been calculated. For task 3 the performance parameters have been calculated for the ascending and descending part of the ramp separately. Steadiness was defined as the standard deviation of the difference between actual torque and regression line. This way fluctuations of the torque could be quantified while time-shifting was neglected (Figure 2). To quantify, if despite fluctuations the dictated slope has been achieved, the regression line through the torque data has been calculated additionally. A perfect slope was 0%/s for task 2 and 12%/s and −12%/s for the ascending and descending part of task 3, respectively.

#### 2.5.2 Acceptance

After each task, participants were asked to rate the performed task on a scale from 1 to 5 for fun, difficulty and excitement. The scale was visualized on a board with smileys ranging from happy to sad emotions representing scores 1 to 5, respectively.

### 2.6 Statistical analyses

Statistical analyses followed the “per-protocol” principle, i.e., incomplete data sets were discarded. Repeatability of the MVC and the performance parameters steadiness and slope has been tested using the two-way agreement-type intraclass correlation coefficient (ICC). Between-group comparison within the visits has been performed using Mann-Whitney *U*-test with Holm correction for multiple comparisons. The statistical significance level was set to 5%.

## 3 Results

**Figure 3:**
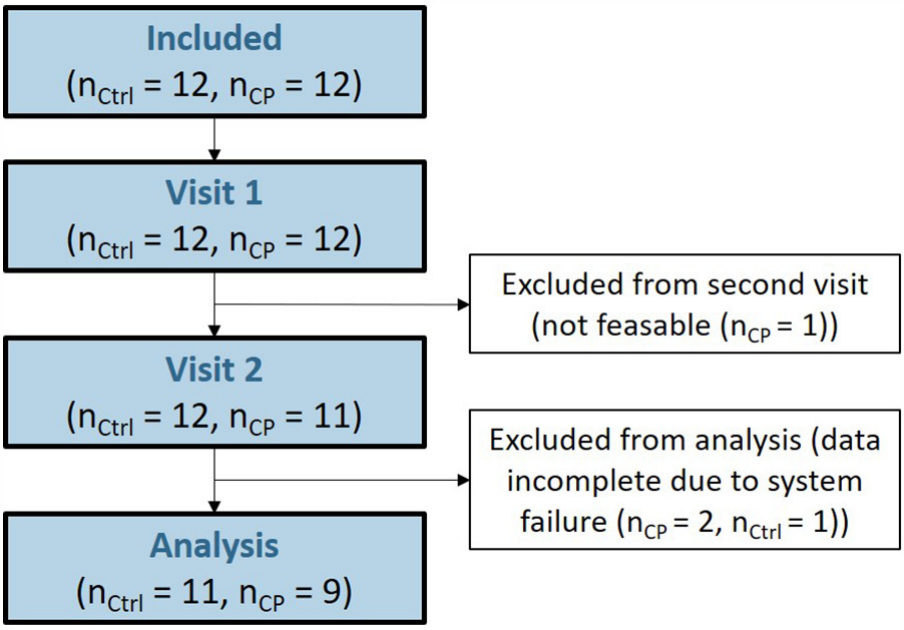
CONSORT-style flow chart.

One participant of the CP group was not able to perform the tasks due to limited cognitive abilities and dropped out for the second visit. All other participants completed both visits successfully. Incomplete data sets were found in two further participants of the CP and one participant of the control group due to failures of the recording systems (Figure 3).

With the remaining 20 participants we found no differences in age, female:male ratio, height and weight between groups (Table 1). Acceptability data collection was stopped after four participants, as these younger children were not able to understand and answer the questionnaire adequately.

**Table 1:** Demographics.

Variables	Group		*p*-value
	Control (*n*=11)	CP (*n*=9)	
Age [years]			0.856
Mean (SD)	12.9 (3.6)	13.2 (3.9)	
Median	13	14	
Range	7–17	8–18	
Sex			1
Female, *n* (%)	3 (27.3)	3 (33.3)	
Male, *n* (%)	8 (72.7)	6 (66.7)	
Diagnose			
USCP, *n* (%)		1 (11.1)	
BSCP, *n* (%)		8 (88.9)	
GMFCS			
II, *n* (%)		2 (22.2)	
III, *n* (%)		7 (77.8)	
Height [cm]			0.298
Mean (SD)	164.0 (22.4)	154.8 (15.9)	
Median	166	155	
Range	126–193	132–175	
Weight [kg]			0.415
Mean (SD)	53.5 (20.9)	46.3 (17.7)	
Median	57	42	
Range	24–85	26–76	

SD, standard deviation; CP, cerebral palsy; GMFCS, gross motor function classification system.

### 3.1 Feasibility

**Figure 4:**
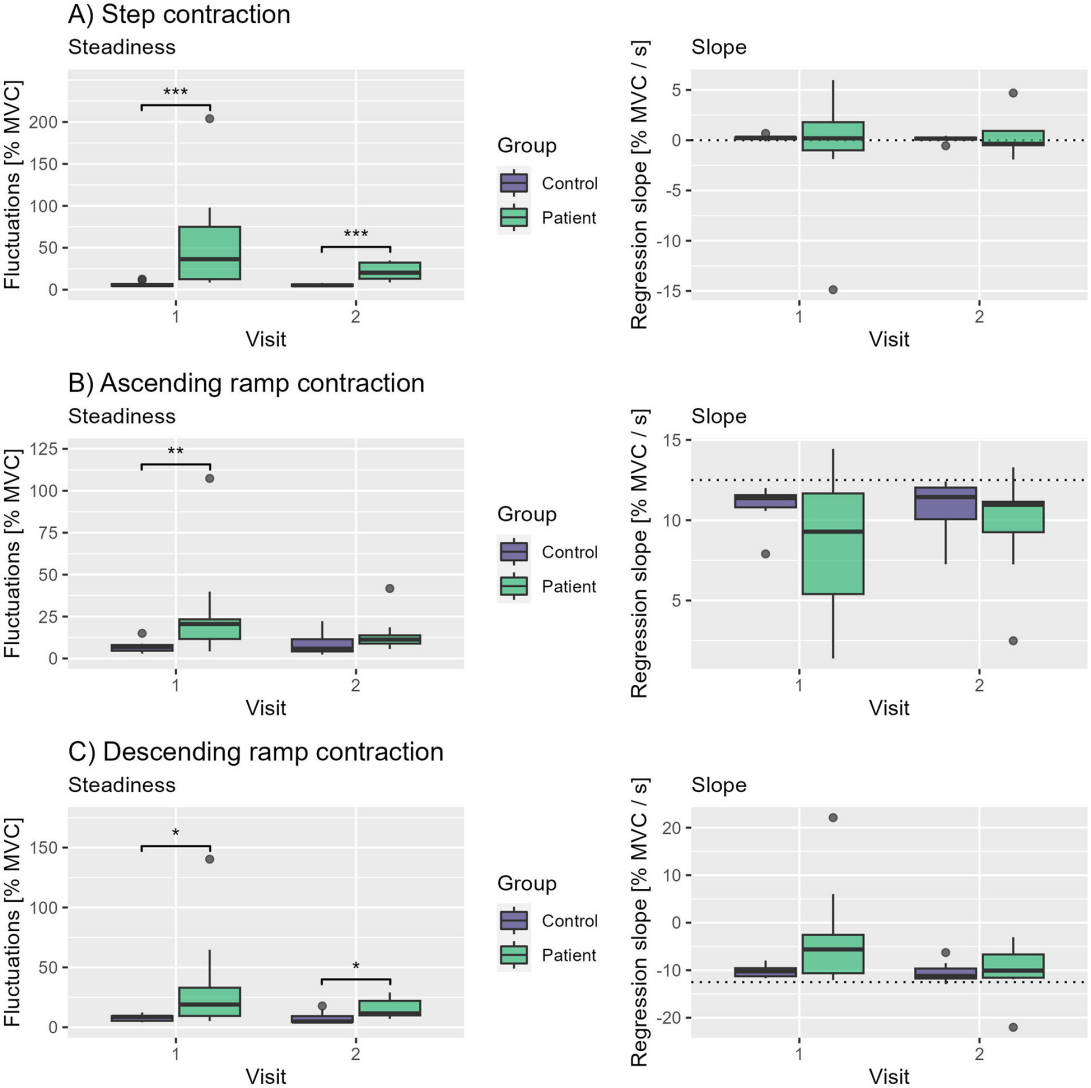
Boxplots of steadiness and slopes of both groups and visits during **(A)** step contractions, **(B)** ascending ramp contractions and **(C)** descending ramp contractions. Significance markers: * <0.05, ** <0.01 and *** <0.001.

During the first visit, participants of the CP group reached a MVC of 1.0 g while the control group reached 4.3 g (p<0.001). Similar results have been found for visit two (1.4 g vs. 4.9 g, p<0.001). Plantarflexion torque fluctuated more in the CP group in all tasks at visit 1. During step contractions CP group’s torque fluctuated by 52.2% MVC vs. 6.1% MVC in the control group (p<0.001). Similar differences could be seen during ascending ramp contractions (26.3% MVC vs. 7.0% MVC, p=0.005) and descending ramp contractions (34.1% MVC vs. 8.1% MVC, p=0.025). At visit 2 group differences in steadiness decreased but remained significant for step contractions (20.6% MVC vs. 5.3% MVC, p<0.001) and descending ramp contractions (15.9% MVC vs. 7.7% MVC, p=0.025) but vanished for ascending ramp contractions (Figure 4). There were no significant differences between groups in slopes during all tasks and visits.

### 3.2 Repeatability

**Figure 5:**
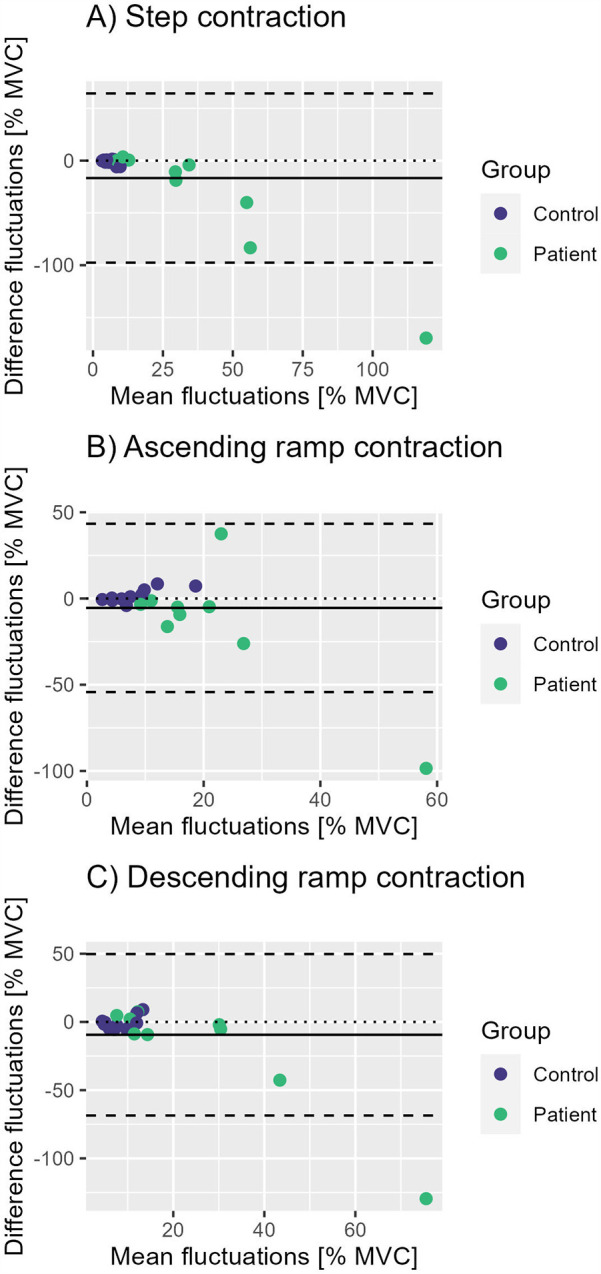
Bland-Altman plots for repeatability of steadiness during **(A)** step contractions, **(B)** ascending ramp contractions and **(C)** descending ramp contractions. The solid line represents the mean fluctuations of all participants, the dashed lines mean ± 1.96 standard deviations.

MVC showed excellent repeatability between the measurements with an ICC of 0.94 (p<0.001), whilst all performance parameters of all tasks showed poor ICCs (Table 2). Consistent with the previously mentioned better performance of the control group during visit 1 and 2, we see only little differences in fluctuations between visits in this group (Figure 5). By contrast, almost all participants of the CP group improve their performance during step and ramp contractions from visit 1 to visit 2 (Figures 5B–D). Despite the lack of any significant group differences between slopes in both visits, there is a visible trend in all tasks of the group to converge (Figure 4).

**Table 2:** Between-visits analysis of MVC and performance parameters.

Parameter		ICC (95% CI)	F-value	df1	df2	*p*-value
MVC (g)		0.94 (0.65–0.98)	57.5	19	4.41	<0.001
Step contraction	Steadiness	0.28 (−0.12–0.63)	1.88	19	1.88	0.085
	Slope	−0.40 (−0.76–0.07)	0.45	19	18.8	0.954
Ascending ramp contraction	Steadiness	−0.00 (−0.44–0.43)	0.99	19	19	0.508
	Slope	0.20 (−0.28–0.59)	1.47	19	19.1	0.203
Descending ramp contraction	Steadiness	0.13 (−0.29–0.52)	1.31	19	19.7	0.277
	Slope	0.14 (−0.26–0.52)	1.35	19	19.9	0.254

## 4 Discussion

Application of our Space-gamified muscle testing protocol turned out to be feasible in most participants and in most of its aspects. A crucial prerequisite to perform successful measurements were good cognitive skills and at least one familiarization visit. Although quantification of acceptance was not feasible with the used questionnaire, subjectively all participants accepted the measurement procedure exceptionally good and voluntarily repeated the testing for visit 2. While MVC testing was feasible and repeatable, the performance of the ramp and step contraction improved during visit two in the CP group. Contrasting with step contractions and decreasing ramp contractions, the performance of children with CP during ascending ramp contractions improved even to the level of control subjects.

This learning effect limits the application of the procedure to investigate interventions. With one familiarization visit, however, the ascending ramp contraction can be applied as measuring method during a second visit. As the measurement setup is at the moment, it requires multiple devices (Game, EMG, Dynamometer, Ultrasound, Trigger) at the same time which makes a rather rapid and easy execution difficult. However, a reduction to just the game run by the dynamometer signal is relatively simple and could be implemented in a clinical setting. A measurement system of choice could potentially be added as needed. The game itself applied to another dynamometer would even allow for measurements of other muscle groups.

The learning effect also shows that the procedure has therapeutic potential to improve contraction control in patients with CP. Especially the gamification of the measurement could be particularly motivating, as gaming technologies grow in popularity in neuropediatric rehabilitation to increase patient compliance (15).

The successfully performed ascending ramp contractions can retrospectively be analyzed in regards to different muscle parameters. The recorded EMG signals can be used to investigate muscle activation and co-contraction from 0% to 100% MVC. From the simultaneously recorded ultrasound sequences architectural parameters like fascicle length, pennation angle and muscle thickness can be extracted to gain insight into the muscle’s mechanics. In future studies the successful application of testing ascending ramp contractions makes additional analyses possible. In 2021 Schranz et al. investigated, inter alia, muscle-tendon-junction displacement to draw conclusions about Achilles tendon lengthening and stiffness. An application of this method during slow and continuous ascending ramp contractions could improve the method, because the slowed down contraction allows higher resolution ultrasound imaging. Ramp contractions could also be used to apply quick release protocols.

In the next step the collected data of muscle activation and architecture will be analyzed retrospectively for the evidentially feasible ascending ramp contraction to define characteristic muscle parameters of patients with CP. Additionally, a currently running follow-up study applies the feasible assessment of MVC and ramp contractions to investigate the effects of whole-body vibration training with 20 subjects and three visits.

## Data Availability

The datasets presented in this study can be found in online repositories. The names of the repository/repositories and accession number(s) can be found below: https://doi.org/10.6084/m9.figshare.27301788.v1.
